# Public Health Research Resulting from One of the World’s Largest Outbreaks Caused by Entero-Hemorrhagic *Escherichia coli* in Germany 2011: A Review

**DOI:** 10.3389/fpubh.2017.00332

**Published:** 2017-12-11

**Authors:** Elena Köckerling, Laura Karrasch, Aparna Schweitzer, Oliver Razum, Gérard Krause

**Affiliations:** ^1^Department of Epidemiology and International Public Health, Bielefeld University, Bielefeld, Germany; ^2^Department Münster, Institute for Rehabilitation Research IfR, Münster, Germany; ^3^Department of Epidemiology, Helmholtz Centre for Infection Research, Braunschweig, Germany; ^4^Hannover Medical School, Hannover, Germany

**Keywords:** disease outbreaks, Shiga-toxigenic/entero-hemorrhagic *Escherichia coli*, hemolytic uremic syndrome, Germany, research

## Abstract

In 2011, Germany experienced one of the largest outbreaks of entero-hemorrhagic *Escherichia coli* (EHEC) ever reported. Four years thereafter, we systematically searched for scientific publications in PubMed and MEDPILOT relating to this outbreak in order to assess the pattern of respective research activities and to assess the main findings and recommendations in the field of public health. Following PRISMA guidelines, we selected 133 publications, half of which were published within 17 months after outbreak onset. Clinical medicine was covered by 71, microbiology by 60, epidemiology by 46, outbreak reporting by 11, and food safety by 9 papers. Those on the last three topics drew conclusions on methods in surveillance, diagnosis, and outbreak investigation, on resources in public health, as well as on inter-agency collaboration, and public communication. Although the outbreak primarily affected Germany, most publications were conducted by multinational cooperations. Our findings document how soon and in which fields research was conducted with respect to this outbreak.

## Introduction

In spring 2011, Germany experienced one of the largest outbreaks of entero-hemorrhagic *Escherichia coli* (EHEC) ever reported, almost 3,000 people fell ill with acute gastroenteritis, 855 of them developed hemolytic uremic syndrome (HUS). In total, 55 people died due to the infection (1). The outbreak affected primarily five states in Northern Germany but also visitors from 15 other countries and was linked to a smaller subsequent outbreak in France (2). In contrast to what would have been expected from previous outbreak reports and surveillance data, this outbreak was characterized by high case fatality, a higher proportion of HUS resulting from the EHEC infection (1.1 cases per 100,000 inhabitants in 2011 versus an average of 0.1 cases per 100,000 inhabitants yearly, in 2001–2010), a predominance of adult patients (3.10 HUS resulting from EHEC infection and 15.5 EHEC cases without HUS per 100,000 inhabitants in 2011 versus less than 0.0 HUS resulting from EHEC infection and EHEC cases without HUS per 100,000 inhabitants yearly, in 2001–2010) and a stronger predominance for female patients (HUS resulting from EHEC infection: 68% female patients, EHEC without HUS: 58% female patients) (1). The outbreak lasted 58 days, from May 8 to July 5, 2011. On May 25, 2011, seven days after the first notification of the outbreak, the causative pathogen was identified to be Shiga toxin (Verocytotoxin)-producing *Escherichia coli* O104:H4, a bacterium with a novel virulence profile in comparison to strains usually prevalent in Europe. From May 20 to July 8, 2011, the Robert Koch Institute (RKI), the German federal public health institute, together with local and state health and food safety agencies, conducted a total of 13 epidemiological field investigations, using different study designs. Initial investigations pointed at lettuce, raw tomatoes, and cucumbers as potential sources of the infection. On June 10—3 weeks after the first notification of the outbreak—epidemiological evidence supported that fenugreek sprouts, produced in Germany from seeds imported from Egypt, were the vehicle causing the outbreak (3). The outbreak resulted in massive challenges for hospitals, public health and food safety agencies, and intense international media coverage. There were significant economic repercussions on the agriculture industry of various European countries, particularly Spain, after the Hamburg secretary of health prematurely presented an unconfirmed laboratory result erroneously implicating cucumber imported from Spain as the potential source of infection (3). This, in turn, had international political and economic consequences, including a temporary embargo of food products exported from the European Union. Although epidemiological studies and investigations supported that fenugreek sprouts were the vehicle, various attempts to detect the pathogen in sprouts consumed by known patients had failed. In early evaluations of the outbreak, Krause et al. (3), Stark et al. (4), and Beutin and Martin (5) highlighted the uniqueness and size of the outbreak. They strongly recommended evaluating the experiences with the outbreak as best as possible in order to better prevent and control comparable situations in the future. In this work, we systematically reviewed the scientific literature related to this outbreak 4 years after its onset. The aim of the study was to trace the scientific process in order to assess to which extent the different disciplines were involved, to identify the collaborations established, and to find out which public health-related topics were researched. The results of this work can help public health authorities to better understand how the scientific community works under the conditions of a disease outbreak. With this knowledge, coordination and collaboration between public health authorities and scientists can be facilitated. This paper also encourages scientists from different disciplines to take a broader view during outbreak investigations, and to contemplate the challenges and potentials of interdisciplinary collaboration.

## Materials and Methods

**Figure 1:**
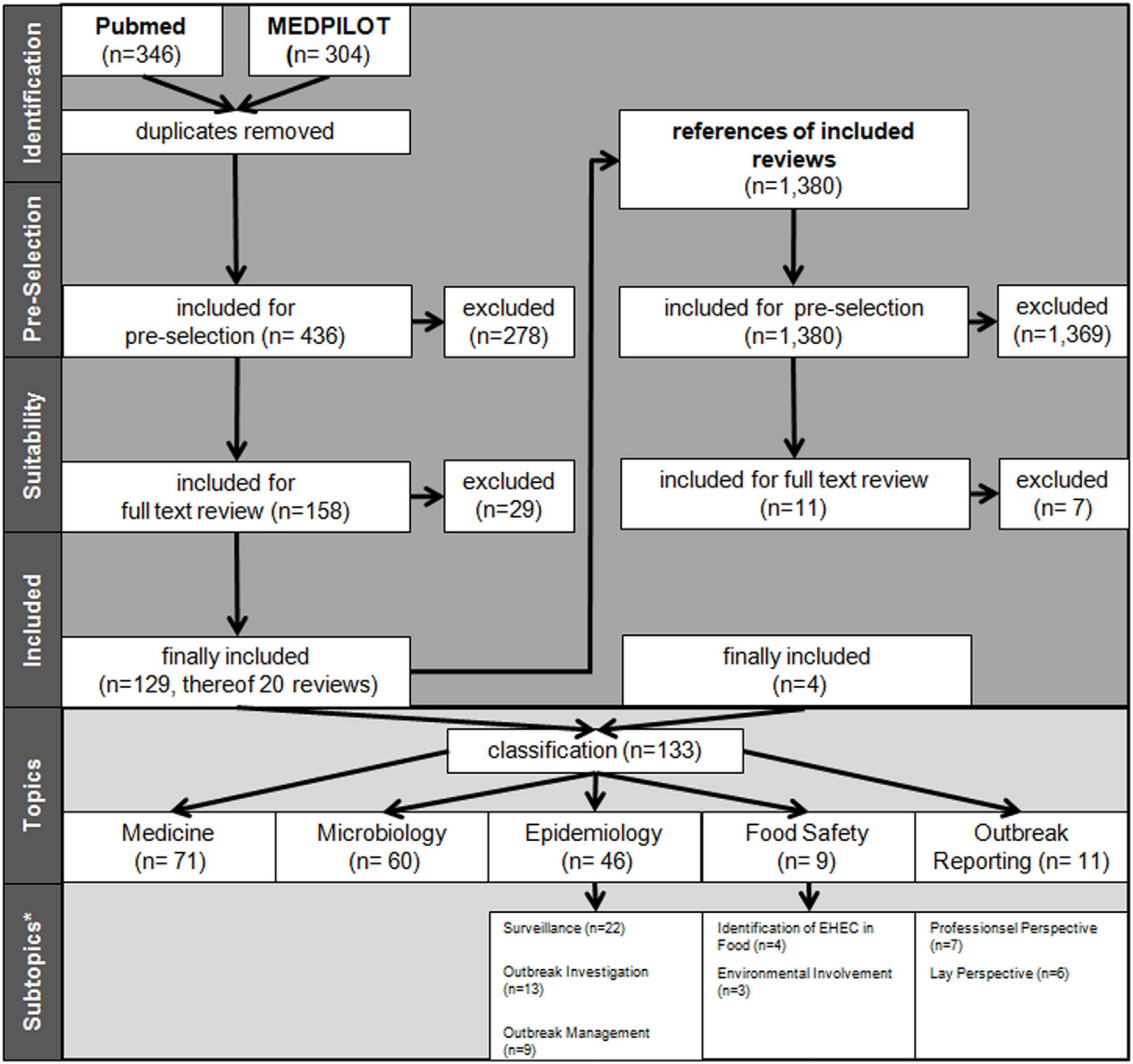
Flow process chart of literature search and selection. *Allocation for subtopics only for other types of articles and not for reviews. More than one topic/subtopic per paper (sums higher than totals).

Following the Preferred Reporting Items for Systematic reviews and Meta-Analyses (PRISMA) guidelines (6), we searched for literature in PubMed and MEDPILOT using the search terms “EHEC” OR “O104:H4” OR “HUS” and their related Medical Subject heading (MESH) “Shigatoxigenic *Escherichia coli*” OR “hemolytic-uremic syndrome” in combination with “Germany” and its MESH term “Germany” appearing in title, abstract or key words for articles published between May 1, 2011 and April 30, 2015. We did not apply any restriction regarding the language of the publications. Since MEDPILOT does not allow retrospectively specifying exact search periods, we initially included publications from January 2011 to April 2015 and later manually excluded those published between January 1 and April 30, 2011. We also included any additional publications that were referenced in retrieved articles and met our inclusion criteria but were not listed in the two databases (Figure 1). We conducted a two-stage screening of the retrieved literature. First, titles and abstracts were screened. Second, eligibility for inclusion in the review was assessed by full-text screening of articles retained after the first screening. Two reviewers (EK and LK) independently screened the literature in duplicate. In the case of discrepant or uncertain results where the two reviewers could not reach consensus, a third reviewer (GK) was consulted. Besides the formal criterion of time of publication, studies had to meet the following criteria for inclusion: they had to be scientific articles published in a publicly accessible journal, but excluding work that was either non-original or not peer reviewed, such as government reports, editorials, commentaries, replies, meeting abstracts, and diaries (e.g., articles reflecting the course of the outbreak in daily intervals from a personal point of view). We also excluded all publications reporting on other outbreaks or strains, as well as publications on the outbreak in question but not constituting original research, such as treatment recommendations not based on research relating to this outbreak. We included articles on cases of O104:H4 infection outside Germany related to this outbreak. We then assigned the articles to one or more of the following pre-defined topics: Epidemiology, Food Safety, and Outbreak Reporting as Public Health topics and Medicine (including disease progression, non-microbiological diagnosis and treatment), and Microbiology (including microbiological diagnosis) as further topics. Contents of publications belonging to the topics medicine or microbiology though were not analyzed, as this would be beyond the public health scope of this review. We also recorded the countries of origin of the authors’ institutions in the categories, “Germany,” “non-German countries,” and international collaborations in the category “Germany and non-German countries.” We assigned each article to one or more of the subtopics listed in Table 1. Additionally, we assigned every article to one of the following three types of publication: review, situation report, and original research. We differentiated between reviews conducted for, or motivated by, the outbreak but not processing data on this outbreak (opportunity reviews); and reviews systematically processing publications on this outbreak (evaluation reviews). Situation reports comprised outbreak reports that were primarily descriptive in nature, while original research only included articles that reported analytical study designs. For the summarized analysis, situation reports and original research were comprised in the category “other publications.” The patterns of respective research activities, namely the publication latency differentiated by topic and the topics differentiated by countries of origin of the authors and by publication type, were assessed for all included publications. For all publications that belonged to the Public Health topics Epidemiology, Food Safety, and Outbreak Reporting, we extracted the main findings and recommendations using a data extraction form developed for this review.

**Table 1:** Definitions of topics and their subtopics.

Topics	Content of the topics	Subtopics	Content of the subtopics
Medicine	– Procedures in medicine – Clinical outcomes, insights gained through autopsy – Long-term outcomes, includes information on shedding and secondary transmission – Stationary and ambulant outbreak management	NA	NA

Microbiology	– Genome analysis – Procedures for genome analysis used or developed for diagnostics with regard to prospective epidemics – Laboratory investigation of the virulence factors and antibiotic resistance – Development of vaccine	NA	NA

Epidemiology	– Cause study – Cases identified *via* the surveillance and other systems – Used or newly developed methods to predict numbers of new cases – Outbreak-related cases outside Germany – Management of the outbreak on federal and regional level and in cooperation with involved institutions	Surveillance	– German surveillance and reporting systems – Asymptomatic cases and secondary cases – Characteristics of the outbreak
		Outbreak investigation	– Investigation of the outbreak source – Methods of food trace back and forward used or invented afterward
		Outbreak management	– Outbreak management, information transfer

Food Safety	– Methods used or newly developed to identify entero-hemorrhagic *Escherichia coli* (EHEC) contamination of food – Environmental involvement (waste water, drinking water, bathing water, ground, etc.) due to direct or indirect (e.g., *via* food production chain) contact to humans	Identification of EHEC in food	– Detection of EHEC in food
		Environmental involvement	– Water as the basis for food production – Animals as a reservoir

Outbreak Reporting	– Information passed on to (professional) public – Methods of risk communication used during the outbreak – Population’s perspective on information coverage – Communication between experts and general population	Professional perspective	– Information released and their content-related arrangement
		Lay perspective	– Scientific investigation on how and what information has been released

## Results

**Figure 2:**
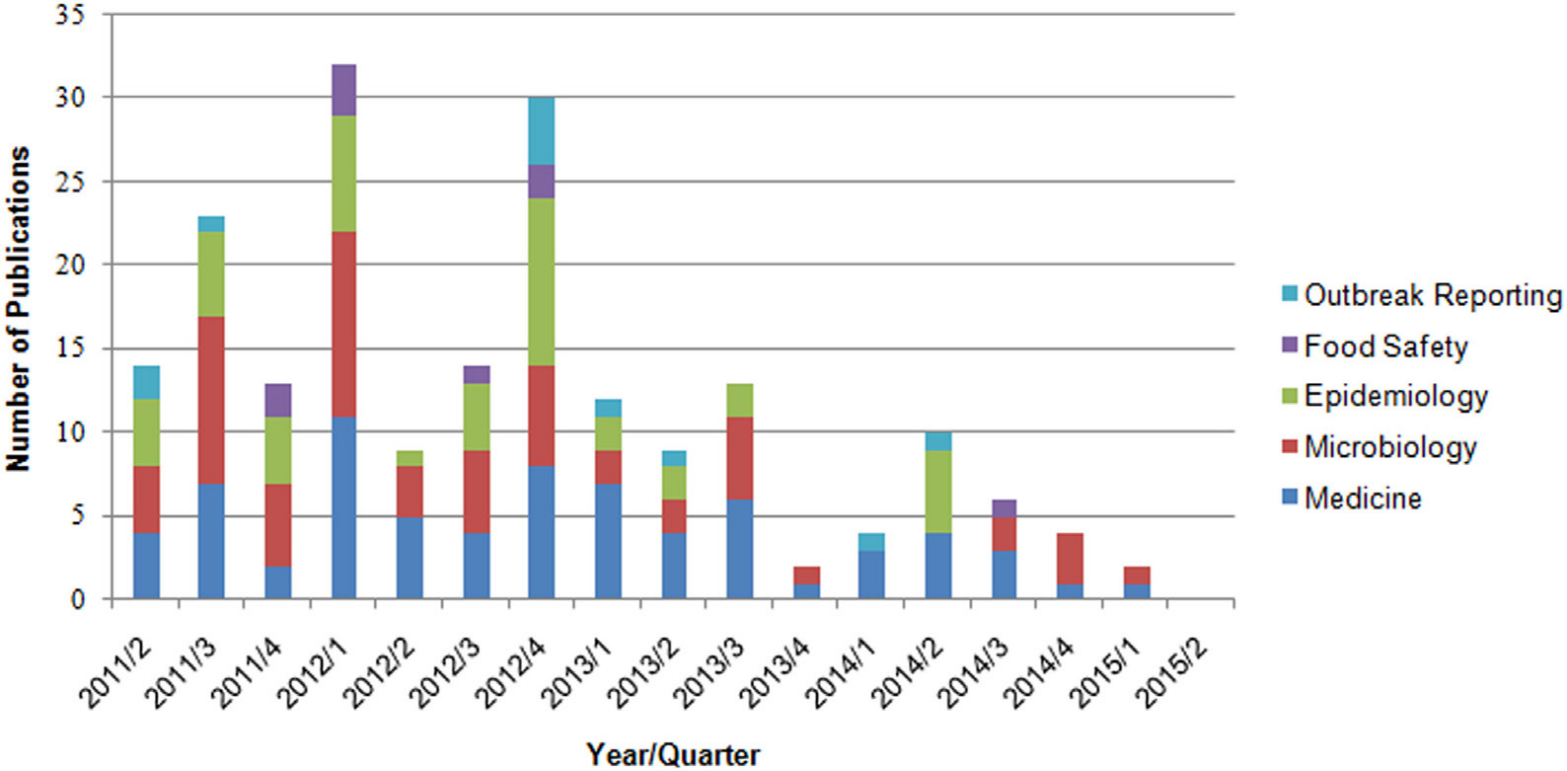
Number of publications by year/quarter and topic (*n* = 133 publications, one paper can be assigned to more than one topic).

By applying the search criteria, we found 346 publications in PubMed and 304 in MEDPILOT; 214 articles (32.9%) were duplicates, resulting in a total of 436 unique publications written in Chinese, Dutch, English, French, German, Polish, Russian, Spanish, or Swedish. Of these, 255 publications were excluded based on content criteria, 47 for being editorials, commentaries, replies, diaries, or meeting abstracts, and five because of being submitted or published before May 1, 2011, leaving 129 (29.6%) after full-text screening. We identified an additional 11 publications on scrutinizing reference lists of eligible reviews, of which four articles fulfilled our eligibility criteria. In total, 133 eligible publications (published in Chinese, Dutch, English, German, and Swedish language) were included in the systematic review (Figure 1; Table 2). Fifty-one articles were published within the first year after onset of the outbreak, 46 in the second year, 19 in the third, and 17 in the fourth year, with a peak of 19 publications in the last quarter of 2012 (Figure 2). In the first year after onset of the outbreak, 20 (24%) publications belonged to the category Epidemiology, ranging between 17 (26%) and 4 (14%) in the following years. Most publications in the first year after the outbreak belonged to the topic Microbiology (30 publications, 37%). The number of publications of this topic dropped to 16 (25%) in the second year and to 8 (29%) in the third year. In the first as well as in the second year after the outbreak, 24 publications appeared within the topic Medicine resulting in 29 and 37%, respectively, followed by 14 publications (50%) in the third year and 9 publications (41%) in the fourth year. As displayed in Figure 2, the topics Food Safety and Outbreak Reporting were the least frequently covered topics throughout the 4-year time period ranging from 6 to 0% for Food Safety and 4 to 8% for Outbreak Reporting. Half of the 133 articles were published within 17 months after the onset of the outbreak (reviews: median 16 months; other publication types: median 17 months). As Figure 2 reveals, we did not find any further literature related to the analyzed topics Outbreak Reporting, Food Safety, or Epidemiology after the third quarter of 2014. Publication latency for all other types of publications was lowest for the topic of Food Safety (median of 9 months) and highest for the topic of Outbreak Reporting (median 20 months). For reviews, publication latency was 11 months in the topic of Microbiology and 18 months in the topic of Medicine (Table 3). The extent of collaboration was determined within the different publication types and topics: International collaboration could be found in 3/20 reviews and in 28/113 other publication types. Most review articles were by institutions from non-German countries only (14/20), while other publication types were mostly by German institutions only (57/113). In total, international collaborations were seen in 31/133 articles. Most articles in the topic Medicine (42/71 = 59%) were exclusively authored by German institutions, whereas articles on Microbiology were mostly authored by non-German authors (32/60 = 53%). In the topic of Epidemiology the predominant authorship setup was German and international collaboration (19/46 = 41%) (Table 4). A total of 39 publications belonged to one or more of the topics Epidemiology, Food Safety, or Outbreak Reporting (Table 4). Their main findings and recommendations are displayed in Table 5. Within the topic of Epidemiology, conclusions and recommendations mainly addressed the following issues: (1) automatization of disease notification and changes in notification laws; (2) methodological improvements in surveillance; (3) personnel resources in public health; (4) factors related to secondary cases; (5) novel outbreak investigation methods; and (6) capacity and collaboration between different agencies and institutions. Within the topic of Food Safety, conclusions and recommendations addressed the issues of (1) sanitation of wastewater; (2) safety of drinking water; (3) methods to detect the pathogens in food/from food samples; and (4) hygienic handling of livestock. Regarding Outbreak Reporting, conclusions and recommendations mainly addressed the question of (1) timing of public information; (2) detail as well as coherence of communication; and (3) coordination of risk communication between different agencies and individuals.

**Table 2:** Publications included in the systematic review (*n* = 133).

Reference	Assigned topics				
	Medicine	Microbiology	Epidemiology	Food safety	Outbreak reporting
(7)		X			
(8)	X				
(9)			X		
(10)	X				
(11)	X		X		X
(12)		X			
(13)		X	X		
(14)	X				
(15)	X				
(16)		X			
(5)	X	X	X	X	
(17)		X			
(18)		X			
(19)	X	X	X		
(20)		X	X	X	
(21)	X	X	X		
(22)	X				
(23)		X			
(24)	X		X		
(25)		X	X		
(26)		X			
(27)		X			
(28)	X				
(29)		X			
(30)	X				
(31)		X			
(32)		X			
(33)	X		X		
(34)			X		X
(35)	X	X			
(36)	X				
(37)	X		X		X
(38)	X		X		
(39)				X	
(40)			X		X
(41)	X		X		
(42)	X	X	X		
(43)	X				
(44)	X				
(45)		X			
(46)		X	X		
(47)		X			
(48)	X				
(49)	X		X		
(50)	X				
(51)	X		X		
(52)	X	X	X		
(53)				X	
(54)		X			
(55)			X		
(56)			X		
(57)	X	X			
(58)	X	X			
(59)		X	X		
(60)				X	
(61)		X			
(62)		X	X		
(63)	X				
(64)	X		X		
(65)	X				
(66)	X				
(3)			X		X
(67)	X		X		
(68)		X			
(69)			X		
(70)	X				
(71)	X				
(72)	X				
(73)			X		X
(74)	X	X			
(75)		X			
(76)	X				
(77)	X	X			
(78)	X				
(79)		X			
(80)	X				
(81)	X				
(82)			X		
(83)	X	X	X		
(84)		X			
(85)		X			
(86)	X				
(87)	X				
(88)		X			
(89)		X			
(90)		X	X		
(91)		X			
(92)	X				
(93)	X				
(94)	X	X	X		
(95)		X			
(96)	X	X			
(97)	X	X	X		
(98)		X			
(99)		X			
(100)			X		
(101)		X			
(102)		X		X	
(103)		X			
(104)	X				
(105)	X		X		X
(106)	X	X			
(107)	X				
(2)		X	X		X
(108)		X			
(109)	X		X		
(110)	X		X		
(111)	X				
(112)		X			
(113)		X			
(114)	X				X
(115)	X				
(116)	X		X		
(117)	X				
(118)		X	X	X	
(4)	X	X	X	X	
(119)	X		X		
(120)				X	
(121)	X				
(122)					X
(123)					X
(124)	X				
(125)			X		
(126)			X		
(127)	X				
(128)	X				
(129)	X				
(130)		X	X		
(131)	X				
(132)		X			
(133)	X				
(134)		X			
(135)	X				

**Table 3:** Median of publication latency in months within the different topics.

Publication type	Topic					
	Medicine	Microbiology	Epidemiology	Food safety	Outbreak reporting	Total
Review	18	11	11	16	NA	16
Other publication types	18	11	18	9	20	17

**Table 4:** Countries of institutions involved by topics.

Countries of institutions		Topics				
		Medicine	Microbiology	Epidemiology	Food safety	Outbreak reporting
Only Germany	Total	42	16	10	4	5
	%	59	27	22	45	46
Only non-German countries	Total	13	32	17	3	4
	%	18	53	37	33	36
Germany and non-German countries	Total	16	12	19	2	2
	%	23	20	41	22	18
All	Total	71	60	46	9	11
	%	100	100	100	100	100

*One paper may cover more than one topic*.

**Table 5:** Structural overview of main findings and recommendations of the extracted publications within the topics epidemiology, food safety, and outbreak reporting (*n* = 39).

Topic, subtopic, issue	Study design	Content	
		Main findings	Conclusions and recommendations
Epidemiology, Surveillance, Standard Process	– Timelines (9) – Evaluation (3, 69) – Cross-sectional study (11, 40, 125)	– Delayed reporting of first cases (3, 9, 69, 125) – Five northern states of Germany were mostly affected (3, 11, 40) – Women are more affected than men (3, 11, 40) – Adults are more affected than children (3, 11, 40)	– Change in the law: development and implementation of an automated notification system (3, 9, 105) – Sentinel surveillance (3, 105)

Epidemiology, Surveillance, Improvement Measures	– Timelines (9, 38) – Evaluation (3, 4, 49, 105) – Cross-sectional study (125)	– Central coordination through the Robert Koch Institute (RKI) (3, 125) – Implementation of daily reporting (3, 9, 125) – Implementation of a syndromic surveillance system (3, 125) – Success depends on the compliance of the involved persons (49) – *E. coli* infections are underestimated through routine surveillance (38)	– Development of an ideal Shiga toxin-producing *Escherichia coli* (STEC) surveillance (4) – Testing all stool samples for enteropathic *E. coli* (38) – 25–30 epidemiologists to support authorities (3)

Epidemiology, Surveillance, Secondary and atypical cases	– Case-control Study (13, 116) – Cross-sectional study (41, 51) – Cohort Study (116) – Laboratory investigation (47) – Evaluation (4)	– Median of shedding was 10–14 days (116) – No secondary infection during post diarrhea (116) – O104:H4 secondary transmission, similar to other Stx 2 types (51) – 33 post-outbreak cases, all caused by secondary transmission (41) – Post-outbreak cases were also tested in France (47) – Proportion of asymptomatic cases is significantly higher among people who consumed sprouts in comparison to people who did not (13)	– Extent of shedding is relevant with regard to secondary cases (4) – Research on the factors influencing the development of clinical symptoms (13) – Testing for the outbreak strain of stool samples from confirmed cases’ household members free of charge enhanced rapid detection of secondary cases (51)

Epidemiology, Surveillance, Cases outside and in Germany	– Cross-sectional study (2, 42) – Case report (59, 67, 83) – Cohort study (64, 109) – Evaluation (3)	– Imported and secondary cases in 15 countries (2, 59, 67, 83, 109) – Mean age 46 years, 71% female (64) – 3,793 cases reported in Germany, 827 hemolytic uremic syndrome (HUS) cases, 53 fatalities (3) – 3,816 cases reported in Germany, 845 HUS cases, 36 fatalities (42) – 88% of the cases are adults (42) – Gastroenteritis cases: median age 46 years, 58% female (3) – HUS cases: median age 43 years, 69% female (3)	– Most of the cases outside the five mostly affected states were travel-related (42) – Web-based case register: used by 73 hospitals in Germany, 17 in Sweden, and 1 in the Netherlands;631 HUS cases were registered (64)

Epidemiology, Outbreak Investigation, General information	– Cross-sectional study (2, 11, 40) – Cohort study (24, 33) – Case-control study (24, 110, 116) – Case report (59, 83) – Trace back and forward (24) – Evaluation (3)	– Information on the outbreak strain (11, 40) – First studies failed to identify the source (3, 33, 59, 83, 110, 116) – Hints on sprouts after fourth case-control study (2, 3, 24) – 13 epidemiological studies conducted by RKI and local authorities (3) – Local authorities declare sprouts from a farm in Lower Saxony as the source, sprouts were withdrawn from the market (3)	– Using data recipes and order lists of restaurant customers to reduce dependence from recall (24) – Search for connections between different events and cases (3, 11) – Many case-control studies failed during the outbreak (33, 110) – Education of catering personal for infectious diseases (33)

Epidemiology, Outbreak Investigation, Food trace back and forward	– Trace back and trace forward (24, 82, 126) – Cohort study (24) – Case-control study (24) – Evaluation (3, 4)	– 41 clusters traced back to sprouts from farm in Lower Saxony (24) – Development of the underlying database took 3 weeks (126) – Information on distribution ways is required (82)	– Developing risk profiles to identify checkpoints in food production chain (4) – Trace back/trace forward should be enhanced as standard method (3, 4)

Epidemiology, Outbreak Management, General information	– Evaluation (3, 69, 105) – Cross-sectional study (2)	– Central coordination through RKI, collaboration with local authorities, WHO, and EU (3, 69) – Special task force implemented, consisted of representatives of federal agencies and the EFSA (3)	– Interdisciplinary collaboration of health and food safety authorities needed (105) – Crisis management to be developed and practiced in advance (69) – Professional associations, Universities and Laboratories to be incorporated into developing crisis management (69) – Information system has to be improved (69) – Education of epidemiologists has to be ensured (69) – Experiences from Germany of use for developing countries (2) – Personnel for interdisciplinary management is needed (3) – Steady communication between clinicians and PH- Service (3)

Epidemiology, Outbreak Management, Information transfer	– Evaluation (3, 73)	– Daily report of the RKI online available (3) – Five joint press conference of the RKI and the Federal Institute for Risk Assessment (BFR) (3, 73) – Over 300 press inquiries answered, 50 TV interviews given by BFR (73)	– Publications should be adjusted between federal and local authorities (3)

Epidemiology, Outbreak Management, Security of supply	– Evaluation (3, 34, 37, 69) – Cross-sectional study (55, 125)	– Lack of information on clinical café capacity during outbreak (3) – Ambulatory treatment in Hamburg and Lübeck was ensured (37) – Clinical care not ensured in 2/79 hospitals in 15/16 German states (125) – Redirecting of information to surgeries was not sufficient (37, 69) – U.S. Department of Defense independently bundled information (34)	– Development of an information system on clinical care capacity (3) – Data consistency is more important than accuracy for case county (55)

Food Safety, Identification of EHEC in food, NA	– Laboratory investigation (60, 102, 120) – Evaluation (4)	– Method based on U.S: Bacteriological Analytical Manual suitable to detect O104:H4 (60) – High-pressure inactivation does not depend on esterase activity (102) – Method based on CHROMagar STEC suitable to detect O104:H4 (120)	– Development of improved methods to identify O104:H4 in food (4) – Reducing *E. coli* shedding of livestock can reduce infection risk for humans (4)

Food Safety, Environmental involvement, NA	– Evaluation (4, 39) – Cross-sectional study (53)	– No evidence of O104:H4 in wastewater (39) – Not conceivable if O104:H4 will establish endemecity in humans or livestock (39) – No infection risk *via* bathing water (53)	– Check on establishment of O104:H4 in livestock or humans (4) – Water used in food production should be of drinking water quality (39) – Development of decontamination methods for wastewater (4) – Development of tests for environmental samples for *E. coli* (4) – Regular testing of drinking water (39)

Outbreak Reporting, Professional Perspective, General Information	–Cross-sectional study (11, 40)	– The BFR gives consumer recommendation and hygiene instructions (11, 40) – Information on research results are given (11, 40)	– Recommendations apply until new evidence is found (11)

Outbreak Reporting, Professional Perspective, Risk communication	– Evaluation (3, 34, 73, 105) – Cross-sectional study (2)	– Inconsistent information was given during the outbreak (2, 3) – Media was used to inform the population (73, 105) – Goals of risk communication were reached (73)	– Information should be objective, correct and not inconsistent (34, 105) – To communicate information on cause, impact, and measures (105) – Information not to be withheld until validity is ensured (73) – Avoiding inconsistent information (3, 73)

Outbreak Reporting, Lay Perspective, Perceived Information	– Evaluation (73) – Cross-sectional study (123)	– Information given by the BFR perceived to be sufficient, – suspension of previous recommendation perceived to be understandable (73)	– Risk communication should be easily accessible (123)

Outbreak Reporting, Lay Perspective, Sources of information	– Cross-sectional study (2, 114, 123) – Evaluation (37, 73) – Cohort study (122)	– TV is perceived to be trustable (73) – Dramatizing contributions in TV fan fear (2, 114) – Web 2.0 for information search perceived to be unsatisfying due to inconsistent information (122) – Capability to reduce personal risk by following recommendation is perceived to be high (123)	– Precise information for the population is needed (37) – Economic loss could have been avoided through better information of the public (123)

## Discussion

We found a total number of 133 original publications directly related to one geographically and temporally rather confined outbreak. Although major publications appeared within days and weeks of the onset of the outbreak (11), the median latency of publications was 17 months with 17 papers (13% of the total) appearing as late as in the fourth year. This indicates that much of the research conducted with respect to this outbreak went beyond immediate outbreak investigations. It addressed research questions that are of relevance for food safety, surveillance, and outbreak investigation methods in general, but also relate to clinical and microbiological aspects of EHEC. This is further supported by the broad spectrum of topics and subtopics covered by these publications. We identified some characteristic differences among topics not only with respect to publication latency but also with respect to the composition of institutions contributing to the publications (Table 3). As expected, the majority of the articles were published exclusively by, or led by, German institutions. However, given that the outbreak primarily occurred in Germany, the proportion of non-German collaborators or lead authors appears to be high. Non-German participation or lead authorship was most common in Microbiological research on the causative *E. coli* strain and in reviews based on prior publications. This is easily explained by the fact that these kinds of publications did not require active involvement in the epidemiological investigation or clinical patient management (19, 118). This may also explain the somewhat unexpected observation that reviews tended to be published earlier than other types of publications (Table 3): while clinicians and public health agents were primarily occupied with managing the outbreak and later generating and analyzing the data derived from it, non-involved (and thus more likely non-German) specialists in the field could without any delay focus on analyzing the existing literature. Such division of tasks appears rational and useful, since early review on specific management questions could help those primarily involved in the management to adapt their interventions. Similarly, laboratories outside the primarily affected country can complement the microbiologic research related to the outbreak once an isolated strain is made available, thus supporting local laboratories to rapidly characterize the implicated pathogen. The EHEC outbreak was exceptional in many aspects and provided a unique opportunity for better understanding the disease (5). Some of the findings published on the outbreak may appear redundant (Table 5). This does not necessarily signify duplicate publication, since arriving at similar conclusions using different study populations or diverse analytic methods applied to the same study population is a desired process in science. It is also justified in an acute outbreak situation to quickly publish results of early analyses and later complement them with more complete or methodologically refined studies (73). We found many case reports, particularly in the field of Medicine. Due to their specific methodological limitations, individual case reports have a limited potential to contribute significantly to pressing research questions, unless a pooled analysis of cases is performed. Unfortunately, no such analysis seems to have been done so far. Our search strategy including two independent data bases and all languages is likely to have led to a rather comprehensive collection of publications. We believe our decision to exclude editorials, commentaries, replies and diaries, is unlikely to result in relevant original research findings to be missed. Furthermore, this approach ensured that only scientifically reviewed publications were included and it inhibited dissemination of politically motivated communications by stakeholders or interested parties. Nevertheless, larger and more comprehensive studies on the outbreak may still be published in the future, especially in the field of Medicine. The intention to address the patterns of respective research activities from diverse disciplines resulting from this outbreak, instead of addressing one specific research question, precluded us from conducting a meta-analysis. We limited data extraction to Public Health-related topics as we wanted to focus on the scientific output generated in this field. The observed latency of publications on the German EHEC outbreak 2011 highlights the fact that even several years after such an event, original research work continues to be published. We also found a considerable level of cooperation between large number of institutions from within and outside Germany. Our review was not designed to judge the content and scientific merit of these publications. We do not expect that the limitation to 4 years after onset of the outbreak influenced the results of this review as we observed that the number of publications decreased toward the end of the inclusion period. Moreover, novel findings and conclusions were less common in the later years of the observation period. This work reveals a unique map of how the EHEC outbreak was scientifically processed. Nevertheless, the large number of original publications found in our search, and the breadth of topics covered, suggests that the scientific community made appropriate use of this outbreak for research. Compared with the scientific output of the O157:H7 outbreak in the USA in 2009 and in Japan 1996, the EHEC outbreak in Germany has resulted in a higher number of publications. A preliminary search in PubMed indicates that the EHEC outbreak in Germany in 2011 has resulted in approximately four times as many publications as the *E. coli* O157:H7 outbreak in the USA and three times as many as the outbreak in Japan. The toll of the outbreak in terms of morbidity, mortality, and economic losses was undoubtedly high, which may explain the large body of scientific publications in diverse disciplines. The extent to which recommendations resulting from it have actually contributed to improvements in policies and practice merits follow-up research.

## Resource Identification Initiative

[PubMed {SCR:004846}].

## Author Contributions

GK conceived this study. EK led the literature review and conducted the descriptive and content analysis. EK and LK performed the systematic review. All authors (EK, LK, AS, OR, GK) contributed to the study design, interpretation of the data, and to the writing and revision of this paper. All authors agree to be accountable for the content of the work.

## Conflict of Interest Statement

The authors declare that the research was conducted in the absence of any commercial or financial relationships that could be construed as a potential conflict of interest.
